# Urokinase plasminogen activator secreted by cancer-associated fibroblasts induces tumor progression via PI3K/AKT and ERK signaling in esophageal squamous cell carcinoma

**DOI:** 10.18632/oncotarget.15857

**Published:** 2017-03-02

**Authors:** Baoqing Tian, Xiaojia Chen, Huihua Zhang, Xiaoyan Li, Jiakang Wang, Wei Han, Li-Yi Zhang, Li Fu, Yan Li, Changjun Nie, Ying Zhao, Xuan Tan, Hailong Wang, Xin-Yuan Guan, An Hong

**Affiliations:** ^1^ Institute of Biomedicine & Department of Cell Biology, Jinan University, National Engineering Research Center of Genetic Medicine, Guangdong Provincial Key Laboratory of Bioengineering Medicine, Guangzhou, China; ^2^ Cancer Center of Guangzhou Medical University, Guangzhou, China; ^3^ Department of Clinical Oncology, University of Hong Kong, Hong Kong, China; ^4^ Shenzhen Key Laboratory of translational Medicine of Tumor and Cancer Research Centre, School of Medicine, Shenzhen University, Shenzhen, China; ^5^ Sun Yat-Sen University Cancer Center, State Key Laboratory of Oncology in South China, Collaborative Innovation Center for Cancer Medicine, Guangzhou, China

**Keywords:** uPA, CAFs, PI3K/AKT, ERK, ESCC

## Abstract

Cancer-associated fibroblasts (CAFs) are believed to influence tumor behavior and clinical outcomes. We previously showed that conditioned medium (CM) from CAFs induces proliferation and motility of esophageal squamous cell carcinoma (ESCC) cells. Here, we investigated the molecular mechanisms by which the CAF-secreted proteins induce ESCC development and progression. Using antibody arrays, we identified urokinase plasminogen activator (uPA) as one of the main proteins whose release was increased in CAFs compared to normal fibroblasts (NFs). Immunohistochemical analysis of pathological sections showed that uPA-positive cells were localized at the boundaries of tumor and stroma tissues, in stroma between tumor nests, and within the tumors. Increased stromal uPA levels (132/146 cases) correlated with tumor invasion (p < 0.05) and overall survival of ESCC patients (p < 0.05). *In vitro* assays showed that uPA promotes ESCC cell proliferation, migration, and invasion via PI3K/AKT and ERK signaling pathways. *In vivo*, anti-uPA antibody suppressed tumor growth in ESCC xenografts. These results suggest that uPA released from stroma, and especially from CAFs, might be a predictive marker for ESCC diagnosis and prognosis, as well as an effective therapeutic target.

## INTRODUCTION

Esophageal carcinoma (EC) is one of the most common cancers, with a poor prognosis. The major histological subtype of EC is esophageal squamous cell carcinoma (ESCC) [1]. The stroma of a carcinoma is the connective tissue below the basal lamina that directly surrounds the tumor cells [2]. Multiple components, including fibroblasts, extracellular matrix (ECM) and immune cells, form the cancer stroma [3, 4]. Cancer-associated fibroblasts (CAFs) are the activated form of fibroblasts in the tumor stroma [5] and are considered as the predominant cell type of the stroma [6]. Via paracrine manner, CAFs secrete a variety of soluble factors that promote tumor growth and invasion [7], enhance angiogenesis [8], and activate endothelial cells and pericytes [6, 9].

Urokinase plasminogen activator (uPA) is a serine protease that converts plasminogen into the active serine protease plasmin. uPA mediates proteolysis of ECM by binding to a specific cell surface receptor, urokinase plasminogen activator surface receptor (uPAR) [10, 11]. uPA also participates in the initiation of epithelial–mesenchymal transition (EMT), angiogenesis, tumor invasion, and metastasis, as well as in signal transduction pathways [10, 12]. Among breast cancer patients without lymph-node involvement, high levels of uPA correlate with poor prognosis. According to the American Society of Clinical Oncology, uPA may be a key factor in risk assessment and a possible treatment target [13, 14]. In clinics, a strong correlation has been found between cellular levels of uPA and aggressive behavior of ESCC [15–18]. However, the mechanism how uPA contributes to ESCC progression is unknown.

Our previous study has shown that CAFs induce a suitable microenvironment for ESCC development and progression compared with normal fibroblasts (NFs); however, the molecular mechanisms were unknown. In this study, an antibody array was used to identify the effectors released from ESCC-derived CAFs versus NFs that promote tumorigenesis. The results show that the high uPA expression is one of the major differences between CAFs and NFs. uPA derived from CAFs promotes ESCC cell proliferation, migration, and invasion *in vitro*. When uPA is blocked by anti-uPA antibody, the primary tumor growth is suppressed, and PI3K/AKT and ERK signaling pathways are deactivated. Immunohistochemistry analysis demonstrates a high uPA expression in ESSC stroma, including CAFs, had a reverse correlation between uPA levels and overall survival rates.

## RESULTS

### Expression of uPA is higher in CAFs than in NFs

**Figure 1:**
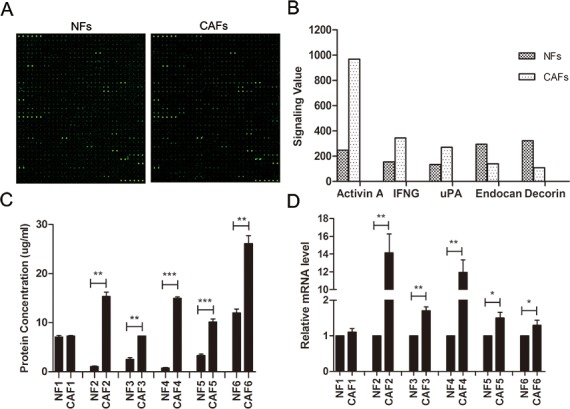
Expression of uPA is increased in ESCC CAFs **A**. The fluorescent figures of antibody array. **B**. Representative images of original values of secreted proteins from CAFs and NFs. **C**. ELISA of secreted uPA in CAFs and NFs (n=6). **D**. qRT-PCR of uPA mRNA levels in CAFs and NFs (n=6). qRT-PCR data were normalized to the housekeeping gene β-actin. Experiments in C and D were done in triplicates. Error bars, mean ± SD.

Antibody array analysis of conditioned medium (CM) was performed from 4 pooled CAFs and their matched NFs, by using the RayBiotech L507 antibody array (Figure 1A). Compared with CM from NFs, 43 up-regulated proteins (8.48%) and 31 down-regulated proteins (6.11%) were detected in CM of CAFs using an arbitrary cutoff line of signal ratio of >1.50 or <-1.50 (Supplementary Table 1). Figure 1B shows the original normalization values of several candidate proteins. Compared to CM from NF controls, the uPA levels in CM from CAFs were more than two times higher. The uPA concentration in CAF CM was confirmed by ELISA; the average uPA concentration in CAF CM was 7.26 times higher than in NFs (Figure 1C). In addition, the uPA mRNA expression in CAFs was 5.28 times higher than in NFs (Figure 1D).

### uPA is expressed in primary ESCC tissues

**Figure 2:**
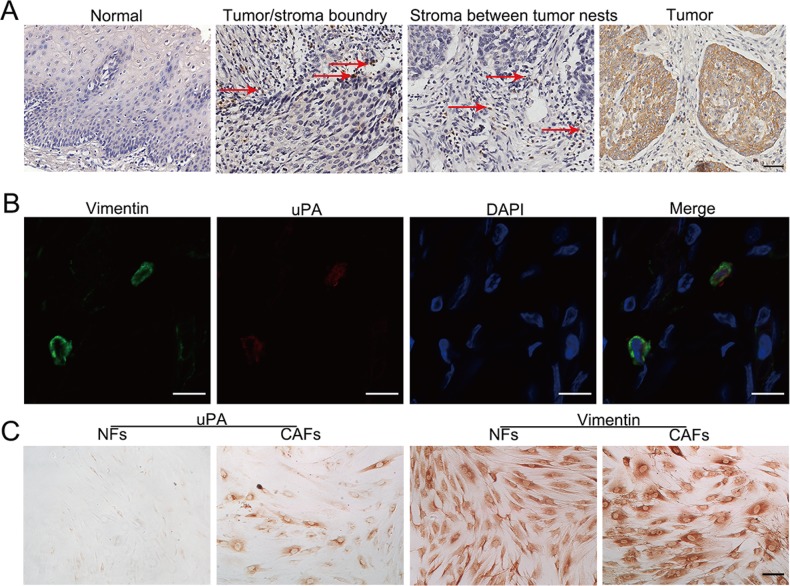
Distribution of uPA positive cells in primary ESCC tissues **A**. Representative image of uPA-positive cells in human ESCC tissue and normal esophagus. In ESCC tissue, uPA-positive cells are frequently observed at the tumor/stroma boundary, in stroma between tumor nests, and within tumor tissue. The arrows indicate uPA-positive cells in tissues. Scale bar 100um. **B**. Representative images of CAFs labeled by antibodies against vimentin (green) and uPA (red). Nuclei were counterstained with DAPI (blue). Scale bar 200um. **C**. Immunocytochemistry with anti-uPA antibody for uPA expression in CAFs and NFs isolated from clinical samples. Vimentin was used as the fibroblast cell specific marker. Scale bar 100 um.

To find out the distribution of uPA-positive cells in primary ESCC tissues, we performed immunohistochemistry (IHC) staining in a panel of tissue microarrays (TMAs) consisting of 300 matched pairs of primary ESCC and non-tumor specimens. Informative results of IHC staining of uPA were collected in 142 primary ESCC cases. Non-informative samples, such as lost samples, samples with too few cells, and inappropriately stained samples, were not used as valid data. The uPA-positive cells were located at the boundary between tumor and stroma tissues, in stroma between tumor nests, and within tumor tissues; however, uPA-positive cells were almost undetectable in adjacent non-tumorous esophageal tissues (Figure 2A). Our previous results have indicated that uPA is increased in fibroblast growth factor receptor 2-positive cells, which is identified as CAFs, but not NFs [7]. We performed tissue double immunofluorescence using two-color (green vimentin; red uPA) staining and confirmed that uPA and vimentin were co-expressed in the same cells, suggesting that the uPA-positive cells were fibroblasts (Figure 2B). We then separated fibroblast cells from the primary tumors and their matched para-tumors, and performed immunocytochemistry. According to the immunocytochemistry results, uPA was expressed in cytoplasm of CAFs, but not in NFs (Figure 2C).

### uPA stroma expression correlates with ESCC invasion and poor prognosis

**Figure 3:**
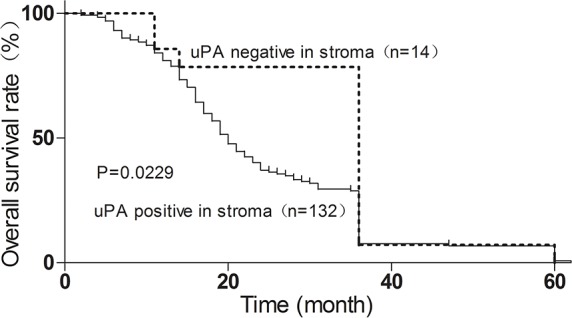
Stroma uPA expression correlates with poor ESCC prognosis, determined by Kaplan–Meier analysis Dotted line, patients with uPA-negative expression (n = 14, median survival 36 months); solid line, patients with uPA-positive expression (n = 132, median survival 20 months; *p<0.05, log-rank test).

IHC analysis revealed that 90% (132/146) of primary ESCC cases were uPA-positive in ESCC stroma. The uPA stroma expression was associated with tumor invasion (Fisher's exact test, P = 0.0393) (Table 1). Kaplan–Meier analysis was used to determine the survival curves of patients with uPA-positive and uPA-negative expression in stroma. The results showed that the overall survival was significantly shorter in patients with uPA-positive stroma expression (n = 132, median survival time 20 months) compared with patients with uPA-negative expression (n = 14, median survival time 36 months) (Figure 3).

**Table 1:** Association of uPA expression levels in ESCC stroma and tumor nest with clinicopathological features in 146 clinical ESCC cases

Clinicopathologic features		uPA in stroma				uPA in tumor nest		
		Total cases (n)	Stroma positive (n)	χ2	P-value	Moderate and high (n)	χ2	P-value
Age	≥57	90	83	0.0450	0.8320	40	0.0138	0.9064
	<57	56	49			24		
Gender	Female	66	58	0.0450	0.8321	32	0.4110	0.5215
	Male	80	74			32		
Depth of invasion	1	8	1			1		
	2	6	1	8.3520	0.0393*	2	1.8620	0.6016
	3	32	32			14		
	4	100	98			47		
T status	T1–2	51	39	0.9186	0.3379	19	0.5506	0.4581
	T3–4	95	93			45		
LNM	N0	78	69	0.0369	0.8476	34	0.0016	0.9680
	N1	68	63			30		
Clinical stage	I	6	1			1		
	II (a, b)	89	83	3.1747	0.2045	40	0.8960	0.6390
	III–IV	51	48			23		

* Statistical significance (*p* < 0.05) is shown in bold.

We have also investigated the uPA expression in tumor tissues of 146 informative ESCC cases. Our results showed no correlation between clinicopathologic features and patients with moderate and high uPA expression in tumor tissues (Table 1). Kaplan-Meier analysis of survival curves indicated that there was no statistical difference in the overall 5-year survival rates between patients with moderate/high uPA tumor expression and patients with negative/low uPA tumor expression (Supplementary Figure 1). Together, these data indicate a reverse correlation between uPA stroma expression and ESCC prognosis.

### uPA secreted by CAFs increases proliferation and migration of ESCC cells

**Figure 4:**
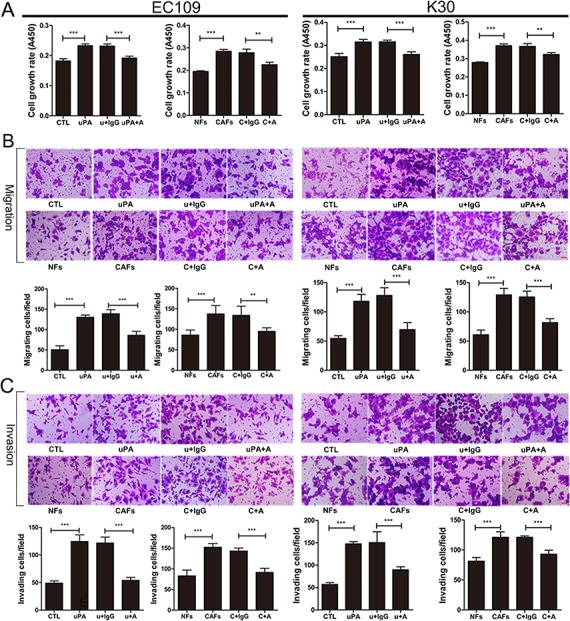
uPA secreted from CAFs functions as oncogenic protein during ESCC progression **A**. Cell growth rates of EC109 and KYSE30. Cells were seeded into 96-well plate at a density of 3×10^3^ per well. After 6 h, cells were treated with either DMEM control or NF CM or 20 ng/ml uPA or CAF CM or 20 ng/ml uPA with 6 ug/ml IgG or CAF CM with 6 ug/ml IgG or 20 ng/ml uPA with 6 ug/ml anti-uPA antibody or CAF CM with 6 ug/ml anti-uPA antibodies. Cell growth rates were compared by WST-8 assays 48 h later. **B**. and **C**. Representative images of migratory and invasive cells per field with indicated treatment. Cells were seeded in the upper compartment at a density of 5×10^4^ per chamber. After 6 h, cells were treated with either DMEM control or NF CM or 20 ng/ml uPA or CAF CM or 20 ng/ml uPA with 6 ug/ml IgG or CAF CM with 6 ug/ml IgG or 20 ng/ml uPA with 6 ug/ml anti-uPA antibody or CAF CM with 6 ug/ml anti-uPA antibodies. Migrated and invaded cells were counted after 36 h. Before the experiments, EC109 and KYSE30 cells were serum-starved for 24 h, acid-washed to remove bound endogenous uPA, and then neutralized. CTL: DMEM control, u+IgG: uPA+IgG, uPA+A: uPA+Anti-uPA antibody, NFs: NF CM, CAFs: CAF CM, C+IgG: CAF CM+IgG, C+A: CAF CM+Anti-uPA antibody. Experiments in A–C were repeated at least thrice. Error bars, mean ± SD. Scale bar 50 um.

The increased uPA mRNA and protein levels in CAFs compared to NFs suggested that the uPA released from CAFs might regulate ESCC cells via a paracrine manner. To analyze the effect of uPA on ESCC tumor progression, we treated ESCC cell lines EC109 and KYSE30 with uPA, or with CAF CM containing high levels of uPA (CAF4). Cells treated with 20 ng/ml of uPA or CAF CM had significantly accelerated growth rates than cells treated with DMEM control or NF CM. After neutralizing uPA with anti-uPA antibody, the proliferation rate decreased compared to cells treated with IgG control (Figure 4A). Moreover, when EC109 and KYSE30 cells were treated with 20 ng/ml uPA or CAF CM, they exhibited increased migration and invasive potential compared to cells treated with DMEM or NF CM. Furthermore, anti-uPA antibody co-incubation with uPA or CAF CM decreased the migration and invasive potential of these cells (Figure 4B, C).

### uPA secreted by CAFs contributes to ESCC progression by activating PI3K/AKT and ERK signaling pathways

**Figure 5:**
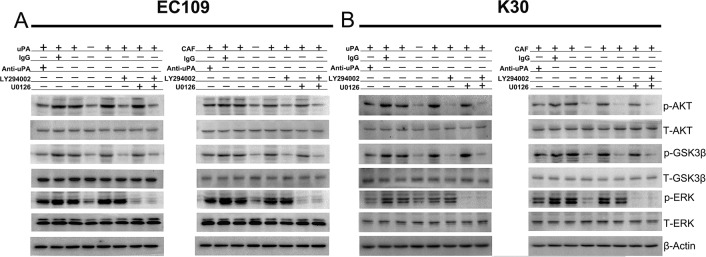
uPA activates PI3K/AKT and ERK signaling pathways in ESCC cells **A**. and **B**. EC109 and KYSE30 cells were incubated in the absence (−) or presence (+) of either DMEM control or NF CM or 20 ng/ml uPA or CAF CM or 20 ng/ml uPA with 6 ug/ml IgG or CAF CM with 6 ug/ml IgG or 20 ng/ml uPA with 6 ug/ml anti-uPA antibody or CAF CM with 6 ug/ml anti-uPA antibody or 20 ng/ml uPA with 10um LY294002 or CAF CM with 10um LY294002 or 20 ng/ml uPA with 10um U0126 or CAF CM with 10um U0126 or 20 ng/ml uPA with 10um LY294002 and 10um U0126 or CAF CM with 10um LY294002 and 10um U0126 for 20 min to assess activation of AKT, GSK-3β, and ERK. In the presence of LY294002 or U0126, cells were pretreated with these inhibitors for 30min. Cell lysates were immunoblotted with anti-phospho- and anti-total-antibodies to AKT, GSK-3β, and ERK. β-actin was used as a loading control. Before all of these experiments, EC109 and KYSE30 cells were serum-starved for 24 h, acid-washed to remove bound endogenous uPA, and then neutralized. Experiments in A, B were repeated at least thrice.

**Figure 6:**
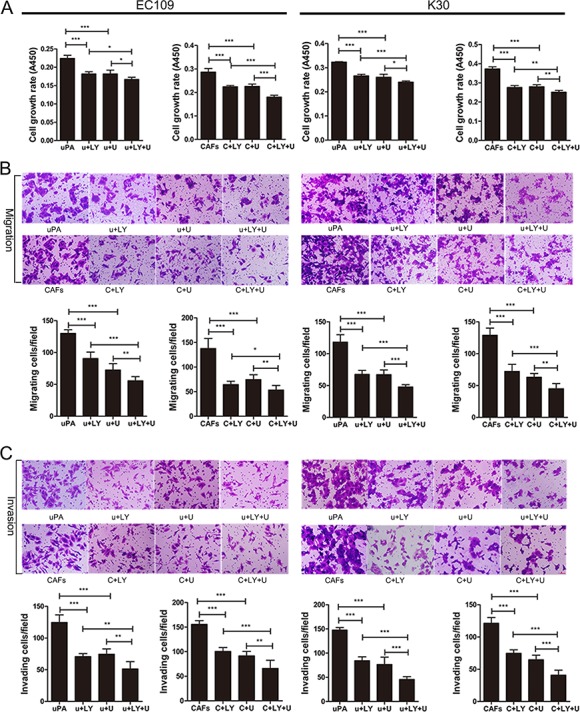
uPA-mediated ESCC progression depends on PI3K/AKT and ERK signaling pathways **A**. Cell growth rates of EC109 and KYSE30. Cells were seeded into 96-well plate at a density of 3×10^3^ per well. After 6 h, cells were treated with 20 ng/ml uPA or CAF CM or 20 ng/ml uPA with 1um LY294002 or CAF CM with 1um LY294002 or 20 ng/ml uPA with 1um U0126 or CAF CM with 1um U0126 or 20 ng/ml uPA with 1um LY294002 and 1um U0126 or CAF CM with 1um LY294002 and 1um U0126. Cell growth rates were compared by WST-8 assays 48 h later. **B**. and **C**. Representative images of migratory and invasive cells per field with indicated treatment. Cells were seeded in the upper compartment at a density of 5×10^4^ per chamber. After 6 h later, cells were treated with 20 ng/ml uPA or CAF CM or 20 ng/ml uPA with 1um LY294002 or CAF CM with 1um LY294002 or 20 ng/ml uPA with 1um U0126 or CAF CM with 1um U0126 or 20 ng/ml uPA with 1um LY294002 and 1um U0126 or CAF CM with 1um LY294002 and 1um U0126. Migrated and invaded cells were counted after 36 h. Before all of these experiments, EC109 and KYSE30 cells were serum-starved for 24 h, acid-washed to remove bound endogenous uPA, and then neutralized. u+LY: uPA+LY294002, u+U: uPA+U0126, u+LY+U: uPA+LY294002+U0126, CAFs: CAF CM, C+LY: CAF CM+LY294002, C+U: CAF CM+U0126, C+LY+U: CAF CM+LY294002+U0126. Experiments in A–C were repeated at least thrice. Error bars, mean ± SD. Scale bar 50 um.

To investigate the uPA-mediated signaling in ESCC cells, we treated EC109 and KYSE30 cells with 20 ng/ml uPA, and analyzed the activity of PI3K, AKT, GSK3β, and ERK1/2. PI3K, AKT, GSK3β, and ERK1/2 were activated during 10–30 min (Supplementary Figure 2A–2D). To investigate whether uPA promotes ESCC progression via PI3K/AKT or ERK signaling pathways, we treated EC109 and KYSE30 cells for 30 min with 10 μM LY294002, PI3K inhibitor, or U0126, MEK inhibitor, before uPA or CAF CM treatment. Our results show that uPA and CAF CM activate AKT, GSK3β, and ERK1/2. When uPA was neutralized with anti-uPA antibody, AKT, GSK3β, and ERK1/2 phosphorylation levels were reduced. When LY294002 or U0126 was added, phosphorylation levels of AKT, GSK3β, and ERK1/2 were reduced; when both inhibitors were combined, their effects were cooperative (Figure 5A, 5B). In the *in vitro* function assays, we added 1 μM LY294002 and/or U0126 to inhibit PI3k/AKT and/or ERK signaling pathway before treating cells with uPA or CAF CM. Proliferation, migration, and invasion rates were significantly weakened in EC109 and KYSE30 cells activated with uPA or CAF CM (Figure 6). However, when EC109 or K30 cells were treated with 1 μM LY294002 or U0126 without uPA or CAF CM treatment, proliferation, migration, and invasion rates were not significantly changed (Supplementary Figure 3). These results indicate that the uPA-mediated ESCC progression depends on PI3K/AKT and ERK signaling pathways.

### Inhibition of uPA suppresses tumor formation *in vivo*

**Figure 7:**
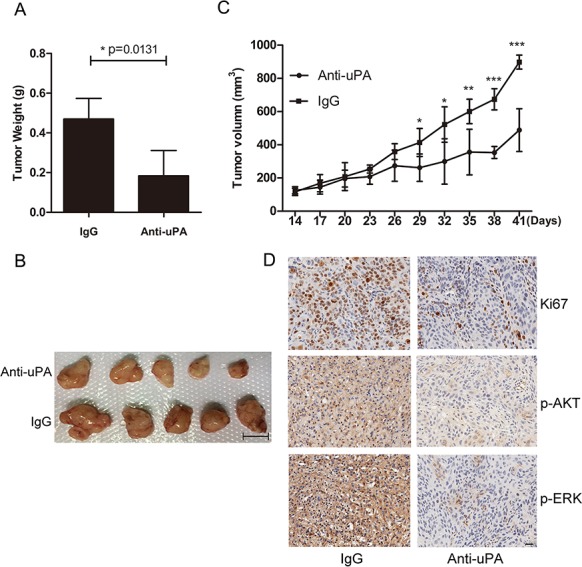
Inhibition of uPA with anti-uPA antibody suppresses tumor formation *in vivo* **A**. Tumor weights in nude mice were compared between mice treated with anti-uPA antibody and control IgG. Error bars mean ± SD. **B**. Representative images of tumors formed in nude mice following injection with anti-uPA antibody or IgG control. Scale bar 1 cm. **C**. Tumor growth curves in nude mice in the two treatment groups. Error bars, mean ± SD. **D**. Representative IHC images of Ki67, p-AKT, and p-ERK in mice treated with anti-uPA antibody or IgG control. Scale bar 50 um.

To investigate the effects of uPA *in vivo*, we used nude mice implanted with KYSE30 cells. Mice bearing KYSE30 tumors (approximately 100 mm^3^) were randomized into two groups (n = 5). One group was treated with control IgG (0.3 mg/kg), and the other was treated with anti-uPA antibody (0.3 mg/kg), by intra-tumor injection every 3 days. After 4 weeks of therapy, the anti-uPA antibody significantly decreased the tumor weight compared with the control IgG group (end-stage tumor weight: 0.1838 ± 0.06376 g in anti-uPA group versus 0.4698 ± 0.05184 g in IgG control group) (Figure 7A, B). The tumor volumes were also significantly reduced in the group treated with anti-uPA antibody compared to the control IgG group (end-stage tumor volume: 488.5 ± 64.36 mm^3^ in anti-uPA group versus 898.3 ± 21.15 mm^3^ in IgG control group) (Figure 7C). Further histologic analysis revealed that anti-uPA antibody reduced tumor protein level of the proliferation marker Ki67, and phosphorylation levels of AKT and ERK (Figure 7D). Quantitative analysis of Ki67, pAKT, and pERK staining is shown in Supplementary Figure 4. These results indicate that the uPA-mediated tumor growth can be arrested by blocking uPA.

## DISCUSSION

Previous studies have indicated that CAFs stimulate tumor growth that provides suitable microenvironment for proliferation, angiogenesis, and invasion of ESCC via secreting various pro-oncogenic factors [6, 8, 19–24]. Most up-regulated genes in CAFs have oncogenic functions, including growth factors FGF18 and HEBGF, transcription factors TFAP2A and TFAP2C, and the Wnt family members WISP1, WNT2, WNT5A, and LEF1. In this study, the antibody array analysis showed that in addition to uPA, CAFs also produce various growth factors and cytokines, such as the TGF-β superfamily (TGF-β3, Activin A, GDF8, and GDF11), VEGF, and interleukins IL-1 and IL-10 (Supplementary Table 1).

Expression of uPA by cancer cells or CAFs depends on the cancer type. uPA is expressed by cancer cells in skin squamous cell carcinoma, whereas in ovarian, breast and colon cancers, it is expressed mainly by fibroblasts [25, 26]. Pyke et al have shown that uPA is localized predominantly in CAFs at the invasive front of tumors in colon adenocarcinoma [27]. In the present study, our results show that CAFs release higher levels of uPA compared to NFs; this is in an agreement with our previous results demonstrating that the uPA mRNA levels are increased in CAFs compared to NFs. We demonstrate that the uPA-positive CAFs are present at the boundary of ESCC tumor and stroma, in the stroma between tumor nests, and within the tumor tissues (Figure 2A). The uPA ESCC stroma levels correlate with the tumor invasion, and negatively correlate with the overall survival rates. In contrast, there is no correlation between pathological features and expression levels of uPA in tumor tissues. However, even in the absence of uPA in tumor tissues, uPA could be still detected in the stroma samples. These findings suggest that it is the uPA expression in stroma, rather than in tumors, that is more directly related to tumor behavior in ESCC.

Previous studies have indicated that uPA activates ERK1 and ERK2 to promote migration of breast cancer cells [28]. In addition, uPA promotes migration of smooth muscle cells by activating PI3K, AKT/GSK3β, and ERK signaling pathways [29]. The down-regulation of uPA inhibits PI3K/AKT-dependent migration of glioblastoma cells [29, 30]. In the present study, *in vitro* analysis showed that uPA secreted from CAFs could induce ESCC proliferation, migration, and invasion, and these promotive functions were dependent on the PI3K/AKT and ERK signaling pathways. Targeting uPA by anti-uPA antibody could effectively neutralize uPA and subsequently deactivate the PI3K/AKT and ERK signaling pathways. These findings provide mechanism of uPA function in ESCC.

The prognosis of ESCC patients is poor and the overall 5-year survival rates range between 15% and 25%. At the time of diagnosis, ESCC is often metastasized to the lymph nodes and distant organs. Therefore, establishing effective detection and treatment strategies for ESCC is essential to improve ESCC diagnosis and prognosis. Our results suggest that uPA secreted from the stroma, especially CAFs, might be used as a predictive marker for ESCC diagnosis and prognosis as well as an effective therapeutic target.

## MATERIALS AND METHODS

### ESCC tumor specimens

CAFs and their matched NFs were isolated from primary ESCC as described previously [31]. Cells were maintained in DMEM medium supplemented with 20% fetal bovine serum (FBS), 100 units/mL penicillin, and 10 mg/mL streptomycin. A total of 26 pairs of ESCC specimens and their matched non-tumor tissues were provided by Guangzhou Medical University Cancer Institute and Hospital (Guangzhou, China). None of the patients received preoperative chemotherapy and radiotherapy. The study proposal was reviewed by Human Subject Research Ethics Committee of Cancer Center of Guangzhou Medical University, and was performed according to the guidelines set forth by this committee.

### Cell lines and reagents

Chinese ESCC cell line EC109 was kindly provided by Professor Tsao (Department of Anatomy, The University of Hong Kong). Japanese ESCC cell line KYSE30 was acquired from DSMZ (Braunschweig, Germany), the German Resource Centre for Biological Material. The cell authenticities were tested by short tandem repeat analysis technology. Cells were maintained in DMEM medium supplemented with 10% FBS, 100 units/mL penicillin, and 10 mg/mL streptomycin. Recombinant uPA protein was bought from Sino Biological Inc. LY294002 and U0126 were obtained from Selleck Chemicals. IgG was purchased from R&D system.

### Preparation of conditioned medium (CM)

CAFs and their matched NFs were seeded into 6-well plates at a density of 2 × 10^6^ per well and grown in serum-free DMEM for 48 h. Culture medium was then collected from each well and centrifuged at 2,000 g for 5 min. The supernatant was collected as conditioned medium (CM) for antibody array and further studies.

### Antibody array

Antibody array (human L507 array, glass slide, RayBiotech) was performed according to manufacturer's protocol. Samples were biotin-labeled and stored at -80°C before the assay. A glass chip was placed in laminar flow hood to dry for 1–2 h. Blocking-buffer was added into each well and incubated at room temperature (RT) for 30 min. Equal volumes of biotin-labeled CM samples from four CAFs (or their paired NFs) were pooled together, and 400 μL of diluted samples was added into wells. Arrays were incubated with gentle shaking overnight at 4°C and then washed briefly; 400 μL of Cy3-conjugated streptavidin was added to each sub-array and incubated at RT for 2 h. The slides were subsequently washed and dried completely before scanning. The data were analyzed with RayBio Analysis Tool software.

### Quantitative real-time PCR

Total RNA was isolated using RNAiso Plus (Takara) according to manufacturer's protocol. First-strand cDNA was synthesized using a first-strand reverse transcription kit (Takara), and qRT-PCR reaction was performed on CFX96 Touch™ Real-Time PCR Detection System with SYBR Green Dye mix (TaKaRa). Primer sequences for qRT-PCR were as follows: β-actin 5′-GCTTGTCCAAGAGT GCATGGT-3′ and 5′-CAGGGCTGGTTCTCGATGG-3′; uPA 5′-CCTGGCACCCAGCACAAT-3′ and 5′-GGGCCGGACTCGTCATAC-3′. Amplification parameters were as follows: 40 cycles of 94°C for 15 s, 60°C for 15 s, and 72°C for 15 s. The results were normalized to β-actin, and calculation was done by comparative C_T_ method (ΔΔ C_T_ method).

### Sandwich ELISA

ELISA was performed using the ELISA kit for uPA (Cloud-Clone Corp). Briefly, 100 μL of diluted CM was added to each well and incubated for 2 h at 37°C. CM was then aspirated, and detection antibody was added and incubated for 1 h at 37°C. Wells were washed, incubated with HRP-linked secondary antibody for 30 min at 37°C, and developed with 3, 3′, 5, and 5′-tetramethylbenzidine.

### ESCC tissue microarray and immunohistochemistry (IHC) staining

A panel of 300 formalin-fixed, paraffin-embedded ESCC tumors and their paired non-tumor specimens was obtained from Linzhou Cancer Hospital. The clinicopathological summaries were collected between 2001 and 2005.

The ESCC tissue microarray (TMA) was constructed as previously described [32]. For IHC analysis, all slides were deparaffinized and rehydrated, and endogenous peroxidase activity was blocked with 3% hydrogen peroxide for 20 min. Antigen retrieval was performed by heating the slides in a microwave oven for 10 min in 10 mmol/L sodium citrate buffer (pH 6.0). The slides were then incubated with goat anti-human uPA polyclonal antibody (R&D system) at 4°C overnight. Subsequently, the slides were incubated with biotinylated donkey anti-goat immunoglobulin for 30 min at 37°C. Isotope-matched human IgG was used in each case as a negative control. Finally, the 3, 30-diaminobenzidine (DAB) substrate Kit (Dako Ltd.) was used for color development followed by Mayer's hematoxylin counterstaining.

The uPA immunoreactivities of the tumor nests and stroma were assessed separately. When five or more uPA positive cells were observed in a 200× microscopy field, it was defined as uPA-positive in ESCC stroma. Scoring of uPA in tumor tissues was performed as follows: (a) proportion of positively stained cells (0, <5%; 1, 6%–25%; 2, 26%–50%; 3, 51%–75%; 4, >75%) and (b) the intensity of staining (0, negative staining; 1, mild staining; 2, moderate staining; 3, strong staining). Scores were measured as a × b; 0 points were defined as negative, 1–3 points were defined as weakly positive, 4–7 points were defined as moderately positive, and 8–12 points were defined as strongly positive. Upon analysis, 0–3 points were considered as uPA-negative and low expression level, and 4–12 points were considered as moderate and high expression level. None of the patients received preoperative chemotherapy and radiotherapy. All clinical samples used in our investigation were approved by the committees for ethical review of research involving human subjects at Zhengzhou University (Zhengzhou, China) and Jinan University.

IHC of mice tumor tissues was performed according to published methods [33]. Ki67 (Sigma-Aldrich), p-AKT and p-ERK (Cell Signaling Technology) positive cells were counted at five arbitrarily selected fields from each tumor at 200x magnification.

### Immunocytochemistry

For immunocytochemistry, CAFs and NFs were seeded into a 96-well plate and cultured in DMEM medium with 10% FBS until 80% confluence. Cells were fixed with 4% paraformaldehyde for 20 min, and the endogenous peroxidase activity was blocked. Antigen retrieval, antibody incubation, and color development were performed as described in the IHC protocol. Mouse antibody against vimentin was purchased from BD Pharmingen.

### Cell proliferation assay

EC109 and KYSE30 cells were starved for 24 h with serum-free DMEM medium, acid-washed (50 mM glycine-HCl, 100 mM NaCl, pH 3.0) for 3 min to remove bound endogenous uPA, and then neutralized (0.5 M HEPES, 0.1 M NaCl, pH 7.5) for 10 min. The effect of uPA on cell proliferation was evaluated by seeding 3×10^3^ cells per well in 96-well plates. After 6 h, cells were treated with 20 ng/ml uPA or CM for 48 h. Proliferation rate was determined by WST-8 2-(2-methoxy- 4-nitrophenyl)-3-(4-nitrophenyl)-5-(2,4-disulfophenyl)-2H-(tetrazolium) assay using a cell counting kit-8 (Dojindo Molecular Technologies) according to manufacturer's protocol. Three independent experiments were conducted.

### Immunofluorescence

For double-labeling immunofluorescence, paraffin block sections of ESCC were incubated with a mouse antibody against vimentin and a goat antibody against uPA simultaneously at 4°C overnight. After brief washing, slides were incubated with Alexa Fluor 594 conjugated donkey anti-goat (Invitrogen) and Alexa Fluor 488 conjugated donkey anti-mouse (Invitrogen) secondary antibodies at RT for 1 h. After brief washing, slides were counterstained with DAPI in anti-fade solution.

### Cell migration and invasion assays

In migration and invasion assays, EC109 and KYSE30 cells were starved, acid-washed, and neutralized as described above. In addition, 5×10^4^ cells were suspended in serum-free medium DMEM and plated in the upper compartment of each chamber (BD Biosciences). For invasion assay, the upper compartments of the chamber were coated with matrigel. The lower compartments were filled with 20% FBS DMEM. After 6 h, cells were treated, and after 36 h of incubation, plates were fixed, stained, and counted under a microscope. Three independent experiments were performed in triplicates.

### Western blot analysis

Western blot analysis was performed as previously described [34]. Densitometry was performed using Quantity One analysis software (Bio-Rad Laboratories). Three independent experiments were performed. The following antibodies were used: β-actin (Sigma-Aldrich), PI3K, AKT, GSK3β, ERK, phospho-PI3K, phospho-AKT, phospho-GSK3β, and phospho-ERK (Cell Signaling Technology).

### *In vivo* mouse model

The *in vivo* function of uPA was investigated by tumor xenograft experiment. Approximately 2×10^6^ KYSE30 cells were injected subcutaneously into the right hind legs of 4-week-old nude mice. When tumors grew up to 100 mm^3^, mice were randomized into anti-uPA antibody (0.3 mg/kg) and control IgG (0.3 mg/kg) groups. Antibodies were injected intra-tumor every 3 days. Tumor growth in nude mice was monitored over a 4-week period. The tumor volume was calculated by the formula V = 0.5 × *L* × *W*^2^ [35]. Animal experiments were performed in compliance with the guidelines for the Welfare of Experimental Animals in Sun Yat-Sen University.

### Statistical analysis

Statistical analysis was performed using SPSS19.0 and GraphPad Prism 5 software. Data are expressed as mean ± SD from at least three independent experiments. Significance was analyzed by Student's *t*-test (two-tailed). The correlation between uPA expression and clinicopathologic characteristics was analyzed using Fisher's exact test. Survival curves were assessed by the Kaplan–Meier method and compared by the log-rank test. Differences were considered significant at *p*<0.05.*, *p* <0.05; **, *p* < 0.01; ***, *p* <0.001.

## SUPPLEMENTARY MATERIALS FIGURES AND TABLES




