# Supplementary material for: Urokinase plasminogen activator secreted by cancer-associated fibroblasts induces tumor progression via PI3K/AKT and ERK signaling in esophageal squamous cell carcinoma
Source: Oncotarget. 2017 Mar 2;8(26):42300–13. doi: 10.18632/oncotarget.15857 (PMC5522068; doi:10.18632/oncotarget.15857)
Supplement: Supplementary file 1 [file oncotarget-08-42300-s001.pdf]

Urokinase plasminogen activator secreted by cancer-associated fibroblasts induces tumor progression via PI3K/AKT and ERK signaling in esophageal squamous cell carcinoma

SUPPLEMENTARY FIGURES AND TABLE

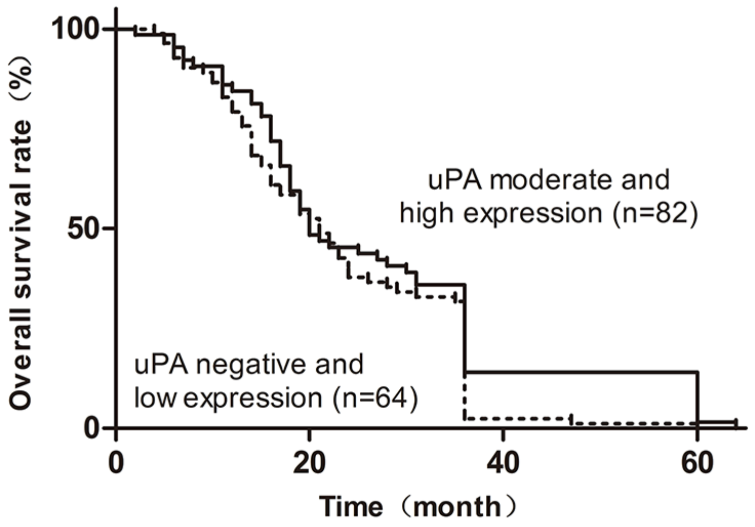

Supplementary Figure 1: uPA expression levels in tumor tissue were not associated with prognosis of ESCC determined by Kaplan–Meier analysis. Dotted line, patients with negative and low uPA expression levels (n = 82, median survival 21 months); solid line, patients with moderate and high uPA expression levels (n = 64, median survival 20 months).

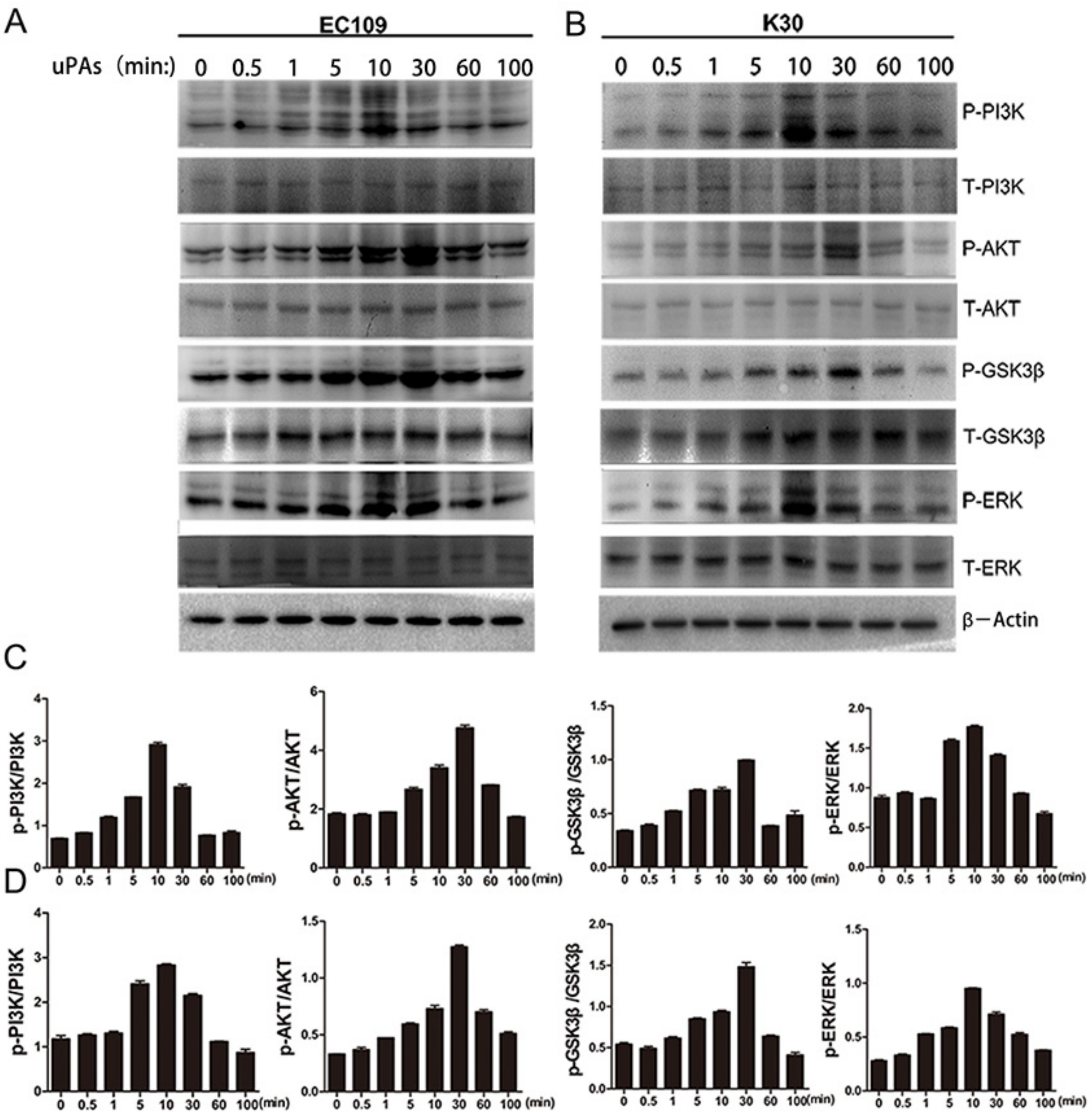

Supplementary Figure 2: uPA activated PI3K/AKT and ERK signaling pathways in ESCC cell lines. A. and **B.** Time courses of PI3K, AKT, GSK3β, and ERK activations in EC109 and KYSE30 cells. Cells were incubated with 20 ng/ml uPA in serum-free medium for different time points: 0, 0.5, 1, 5, 10, 30, 60, and 100 min. Cell lysates were immunoblotted with anti-phospho- and anti-total-antibodies to PI3K, AKT, GSK-3β, and ERK. β-Actin was used as a loading control. **C.** and **D.** The relative phosphorylated ratio of phosphorylation proteins to its total protein, respectively. Before all of these experiments, EC109 and KYSE30cells were serum-starved for 24 h, acid-washed to remove bound endogenous uPA, and then neutralized. T: total; P: phospho. All the experiments were performed at least thrice with similar results.

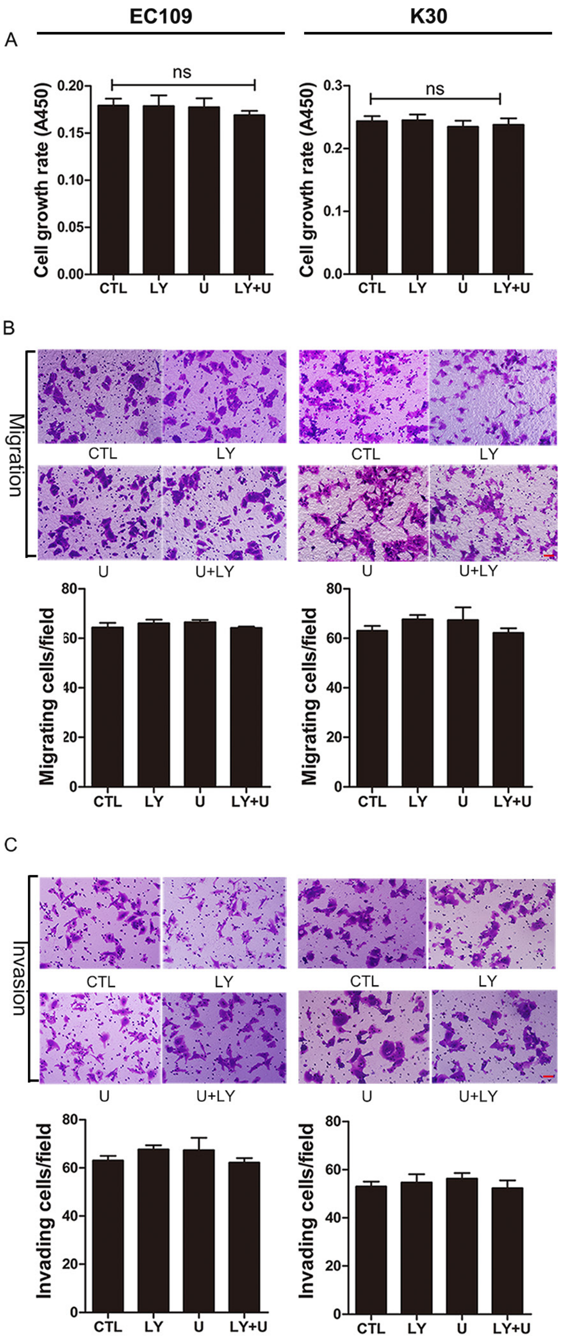

Supplementary Figure 3: LY294002 and U0126 did not affect tumor progression when added without uPA and CAF CM. A. Cell growth rates of EC109 and KYSE30 cells. Cells were seeded into 96-well plate at a density of 3×10^3 per well. Cells were treated with DMEM control or 1 um LY294002 or 1 um U0126, or 1 um LY294002 and 1 um U0126 together. Cell growth rates were compared by WST-8 assays 48 h later. **B.** and **C.** Representative images of migratory and invasive cells per field with indicated treatment. Cells were seeded in the upper compartment at a density of 5×10^4 per chamber. After 6 h, cells were treated with DMEM control or 1 um LY294002 or 1 um U0126 or 1 um LY294002 and 1 um U0126 together. Migrated and invaded cells were counted after 36 h. Before all of these experiments, EC109 and KYSE30cells were serum-starved for 24 h, acid-washed to remove bound endogenous uPA, and then neutralized. CTL: DMEM control, LY: LY294002, U: U0126. Experiments in A–C were repeated at least thrice. Error bars, mean ± SD. Scale bar 50 um.

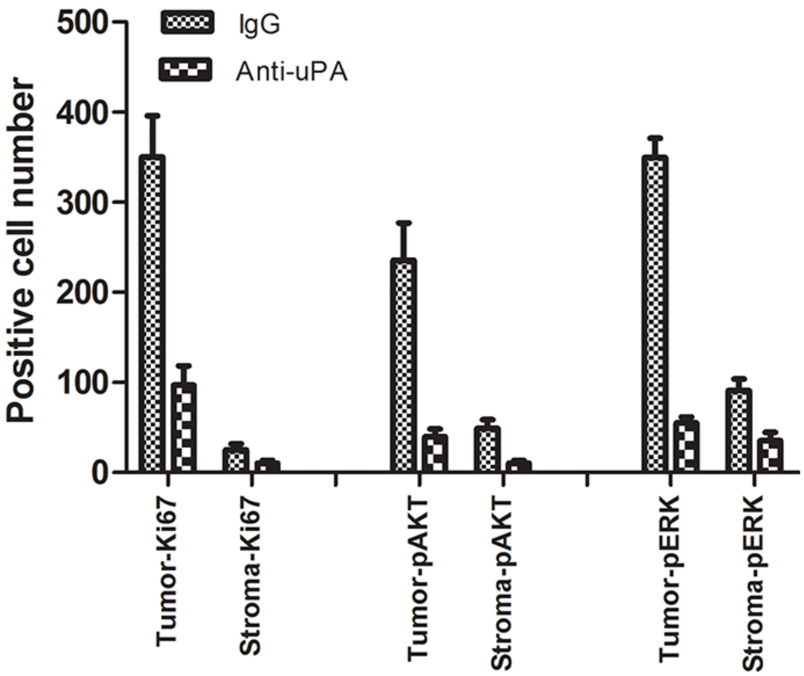

Supplementary Figure 4: Inhibition of uPA with anti-uPA antibody suppressed the expression level of Ki67 and phosphorylated level of p-AKT and p-ERK in mice tumor. After KYSE30 xenograft tissues were analyzed for Ki67, p-AKT and p-ERK levels by IHC, positive staining cells of stroma or tumor tissue were counted respectively at five arbitrarily selected fields from each tumor at 200 x magnifications. Error bars, mean ± SD.

Supplementary Table 1: Secreted proteins profiles by antibody array (fold change > 1.50 or < -1.50).

See Supplementary File 1
