# Supplementary material for: Urokinase plasminogen activator secreted by cancer-associated fibroblasts induces tumor progression via PI3K/AKT and ERK signaling in esophageal squamous cell carcinoma
Source: Oncotarget. 2017 Mar 2;8(26):42300–13. doi: 10.18632/oncotarget.15857 (PMC5522068; doi:10.18632/oncotarget.15857)
Supplement: Supplementary file 2 [file oncotarget-08-42300-s002.docx]

**Supplementary Table S1**

Secreted proteins profiles by antibody array (fold change > 1.50 or < -1.50)

|  |  | **Signaling value** | |  |
| --- | --- | --- | --- | --- |
|  |  |  |  | **NFs/CAFs** |
| **No. of cytokines** | **Cytokines** | **CAFs** | **NFs** | **Fold change** |
| 61 | CD27 / TNFRSF7 | 1 | 92.3 | -90.909 |
| 350 | MIP-3 alpha | 24.5 | 91.28 | -3.731 |
| 367 | MMP-24 / MT5-MMP | 55.5 | 171.79 | -3.096 |
| 504 | VEGI / TNFSF15 | 11 | 33.84 | -3.077 |
| 96 | Decorin | 108.5 | 322.04 | -2.967 |
| 306 | Insulin | 10 | 25.13 | -2.513 |
| 506 | WISP-1 / CCN4 | 11.5 | 28.2 | -2.451 |
| 322 | LIF | 56 | 131.28 | -2.342 |
| 37 | BMP-3b / GDF-10 | 12.5 | 28.72 | -2.299 |
| 223 | IGF-I | 15.5 | 34.36 | -2.217 |
| 472 | TLR4 | 20.5 | 43.59 | -2.128 |
| 111 | Endocan | 139 | 294.86 | -2.123 |
| 355 | MMP-7 | 15.5 | 31.28 | -2.016 |
| 155 | Fractalkine | 12.5 | 25.13 | -2.012 |
| 469 | TLR1 | 27.5 | 53.33 | -1.938 |
| 457 | Thrombospondin (TSP) | 4914 | 9504.68 | -1.934 |
| 113 | Endostatin | 23 | 43.59 | -1.894 |
| 427 | sgp130 | 713.5 | 1308.66 | -1.835 |
| 489 | TSLP R | 14 | 25.64 | -1.832 |
| 501 | VEGF-B | 10 | 17.95 | -1.795 |
| 208 | ICAM-1 | 102.5 | 182.56 | -1.783 |
| 212 | IFN-alpha / beta R1 | 35 | 61.02 | -1.742 |
| 502 | VEGF-C | 51 | 88.71 | -1.739 |
| 330 | Lymphotactin / XCL1 | 10.5 | 17.95 | -1.709 |
| 487 | TROY / TNFRSF19 | 13.5 | 23.08 | -1.709 |
| 465 | TIMP-2 | 220.5 | 371.78 | -1.686 |
| 354 | MMP-3 | 498 | 807.65 | -1.621 |
| 221 | IGFBP-6 | 117.5 | 189.73 | -1.616 |
| 132 | FGF R4 | 36.5 | 58.46 | -1.603 |
| 366 | MMP-20 | 489.5 | 775.35 | -1.585 |
| 266 | IL-12 R beta 2 | 91.5 | 139.48 | -1.524 |
| 41 | BMP-7 | 157 | 104.61 | 1.501 |
| 331 | Lymphotoxin beta / TNFSF3 | 38.5 | 25.64 | 1.502 |
| 171 | GDF8 | 318.5 | 211.78 | 1.504 |
| 193 | GRO | 324 | 215.37 | 1.504 |
| 453 | TGF-beta RII | 69.5 | 46.15 | 1.506 |
| 345 | MIG | 380.5 | 250.76 | 1.517 |
| 321 | LFA-1 alpha | 109 | 71.28 | 1.529 |
| 173 | GDF11 | 363 | 235.89 | 1.539 |
| 227 | IL-1 alpha | 386.5 | 250.76 | 1.541 |
| 432 | Smad 1 | 20 | 12.82 | 1.560 |
| 349 | MIP 2 | 1335.5 | 846.63 | 1.577 |
| 450 | TGF-beta 3 | 28.5 | 17.95 | 1.588 |
| 447 | TGF-alpha | 37.5 | 23.59 | 1.590 |
| 146 | FGF-18 | 117.5 | 73.84 | 1.591 |
| 39 | BMP-5 | 96 | 60 | 1.600 |
| 34 | BLC / BCA-1 / CXCL13 | 84 | 52.31 | 1.606 |
| 259 | IL-10 | 473 | 293.32 | 1.613 |
| 446 | TFPI | 22.5 | 13.85 | 1.625 |
| 203 | HGFR | 462 | 284.09 | 1.626 |
| 191 | Granzyme A | 463 | 280.5 | 1.651 |
| 166 | GCSF | 309.5 | 186.66 | 1.658 |
| 145 | FGF-17 | 135 | 80.51 | 1.677 |
| 378 | Nidgen-1 | 30.5 | 17.95 | 1.699 |
| 340 | MDC | 65 | 37.43 | 1.736 |
| 455 | TGF-beta RIII | 20.5 | 11.79 | 1.738 |
| 317 | LECT2 | 131 | 74.36 | 1.762 |
| 357 | MMP-9 | 32.5 | 17.95 | 1.811 |
| 425 | sFRP-3 | 53 | 29.23 | 1.813 |
| 124 | E-Selectin | 140.5 | 76.92 | 1.827 |
| 210 | ICAM-3 (CD50) | 21 | 11.28 | 1.861 |
| 334 | MCP-1 | 300.5 | 160.51 | 1.872 |
| 480 | TRAIL / TNFSF10 | 39.5 | 21.02 | 1.879 |
| 498 | VEGF | 1832.5 | 952.26 | 1.924 |
| 85 | CXCL16 | 39 | 20 | 1.950 |
| 493 | uPA | 270.5 | 133.33 | 2.029 |
| 402 | PECAM-1 /CD31 | 66.5 | 32.31 | 2.058 |
| 419 | S100A10 | 32 | 15.38 | 2.080 |
| 168 | GDF1 | 176.5 | 80.51 | 2.192 |
| 215 | IFN-gamma | 344.5 | 154.86 | 2.225 |
| 273 | IL-17 | 21 | 9.23 | 2.275 |
| 507 | XEDAR | 10 | 3.08 | 3.250 |
| 2 | Activin A | 967 | 247.68 | 3.904 |
| 106 | EGF | 18.5 | 4.62 | 4.009 |
